# Effects of epidural anesthesia combined with inhalation anesthesia or intravenous anesthesia on intrapulmonary shunt and oxygenation in patients undergoing long term single lung ventilation

**DOI:** 10.12669/pjms.344.14585

**Published:** 2018

**Authors:** Xia Zheng, Zhiquan Lv, Kaiyu Yin, Mingqing Peng

**Affiliations:** 1 Xia Zheng, Department of Anesthesiology, Yongchuan Hospital of Chongqing Medical University, Chongqing, 402160, China; 2 Zhiquan Lv, Department of Anesthesiology, Yongchuan Hospital of Chongqing Medical University, Chongqing, 402160, China; 3 Kaiyu Yin, Department of Anesthesiology, Yongchuan Hospital of Chongqing Medical University, Chongqing, 402160, China; 4 Mingqing Peng, Department of Anesthesiology, Yongchuan Hospital of Chongqing Medical University, Chongqing, 402160, China

**Keywords:** Anesthesia, One-lung ventilation, Oxygenation, Pulmonary shunt

## Abstract

**Objective:**

To investigate the effect of epidural anesthesia combined with inhalation or intravenous anesthesia on intrapulmonary shunt and oxygenation in patients undergoing long term single lung ventilation.

**Methods:**

Eighty patients, aged 35-75, American Society of Anesthesiology (ASA) classification of I-III, undergoing thoracic surgery with one lung ventilation more than three hour, were randomly divided into propofol group (group Pro), propofol combined with epidural anesthesia group (group Pro+Epi), isoflurane group (group Iso) and isoflurane combined with epidural anesthesia group (group Iso+ Epi), 20 patients in each group. Arterial blood and mixed venous blood were taken for blood gas analysis, and hemodynamic data were recorded at following time points: before induction in supine position (T_1_), 30min after bilateral lung ventilation (T_2_), 15min after one lung ventilation (T_3_), 30min after one lung ventilation (T4), 60min after one lung ventilation (T5), 180min after one lung ventilation (T6), intrapulmonary shunt (Qs/Qt) was calculated according to the correlation formula.

**Results:**

Qs/Qt values at T_2-6_ in four groups were significantly higher than that of T1, and Qs/Qt values at T_3-6_ was significantly higher than that of T_2_ (*P*< 0.05); PaO2 at T_2-6_ were significantly higher than that of T1, with PaO2 at T_3-6_ were significantly lower than T_2_ (*P*< 0.05). Between groups, Qs/Qt values in group Iso were significantly higher than that of group Pro, Pro+Epi and Iso+Epi at T_3-5_ (*P*< 0.05). There was no significant difference in PaO_2_ between groups (*P*> 0.05). CI at T3-6 in group Iso and Iso+Epi were significantly higher than that of T_1_ (*P*<0.05), and were significantly higher than that of propofol group (*P*<0.05). MAP at T_3-6_ in group Pro+Epi and Iso+Epi were significantly lower than that at T_1_ (*P* <0.05). Heart rate at T_4-6_ in group Iso were significantly higher than T_1_, and higher than group Pro and group Iso+Epi (*P* <0.05).

**Conclusion:**

One lung ventilation may predispose to increase of intrapulmonary shunt and decrease in arterial partial pressure of oxygen; isoflurane inhalation anesthesia is more likely to cause intrapulmonary shunt, but no changes in arterial partial pressure of oxygen.

## INTRODUCTION

Thoracic surgery often requires one lung ventilation, which may lead to increase in intrapulmonary shunt, decrease of PaO_2_.^1^,^2^ Hypoxic pulmonary vasoconstriction (HPV) is a protective and self regulating mechanism of the pulmonary circulation system in hypoxia, which can greatly reduce the proportion of the pulmonary blood flow to the cardiac output, and improve the pulmonary blood flow under ventilation, and stabilize the PaO_2_.^3^,^4^ HPV can be influenced by various factors such as different anesthesia and drugs,^5^ however, there is limited robust information about how HPV regulate itself independently and pathological changes of one lung ventilation up to three hour, especially under different anesthesia. In this study, we investigated the effects of intravenous and inhalation anesthesia combined with epidural anesthesia on intrapulmonary shunt and arterial oxygenation in patients undergoing long term one lung ventilation.

## METHODS

This study was approved by the ethics committee of Yongchuan Hospital of Chongqing Medical University, and was carried out only after informed consent was obtained from patients and their families. According to relevant statistical and previous data, a prior power calculations showed that 20 patients would be required in each group to detect a 20 percents difference in the SRS with α of 0.05 and β of 0.2. So a sample size of 80 was planned. At last, a total of 80 patients scheduled for elective thoracic surgery under general anesthesia with one lung ventilation up to 3 h were enrolled from our hospital. All patients, aged 35-75 years, with a body mass index (BMI) between 30-50 and an American Society of Anesthesiology classification of I-III, were randomly divided into propofol group (group Pro), propofol combined with epidural anesthesia (group Pro+Epi), isoflurane group (group Iso) and isoflurane combined with epidural anesthesia group (group Iso+ Epi), 20 patients in each group. Patients with severe organ disease, FEVl/FVC<65% and hemoglobin <100 g/L, were excluded.

Patients were randomized to group Pro, group Pro+Epi, group Iso and group Iso+Epi. Randomization was performed using the sealed envelope system, with random treatment assigned to each subject. While in the operation room, monitoring of certain basic vital parameters was provided including non-invasive measurement of arterial blood pressure, pulse oxygen saturation and electrocardiogram, together with ICON (AESCULON, Osypka Medical GmbH, Germany) to monitor the cardiac output. Invasive arterial pressure was performed by radial artery puncture placement under local anesthesia plus 2 mg midazolam for sedation.

Established venous channel, patients received Polygeline Injection 12 ml/kg for volume expansion, after which epidural catheter was placed through the T6-7 space in the left lateral decubitus position. Following intravenous injection of sufentanil 0.4 ug/kg, propofol 1-1.5 mg/kg and 0.1 mg/kg vecuronium for anesthesia induction, a left-sided 37 or 39 Fr Mallinckrodt® (Coviden, Mansfield) double lumen endotracheal tube using standard technique with direct laryngoscopy was placed,^6^ with fiberoptic bronchoscopy showing right main stem intubation. Ventilator parameters were adjusted with tidal volume 8-10ml/kg, respiratory rate was 12 breaths /min, I:E was 1:2, to maintain end-tidal CO_2_ (ETCO_2_) in the rage of 35-45 mmHg. The position of the double lumen tube was confirmed again by bronchoscope before the single lung ventilation in the lateral decubitus position, after which the ventilator parameters were changed with tidal volume 6-8ml/kg, respiratory rate 12-18 breaths/min, I:E 1:1.5, to maintained ETCO_2_ 35-45 mmHg.

During the procedure, patients in group Pro received propofol 100-200 ug/kg/min, cisatracurium 0.1 mg/kg/h, intermittent sufentanil 0.1 ug/kg to maintain anesthesia; for epidural administration, the first dose of 7 ml 0.9% saline, followed continuous infusion of 0.9% saline at rate of 5 ml/h. In group Pro+Epi, same intravenous anesthetic agent plus epidural 0.5% ropivacaine, with first dose of 7ml and followed by a rate of 3-5 ml/h continuous infusion. In group isoflurane, 2-3% inhalation isoflurane was provided together with intermittent sufentanil 0.1 ug/kg, combined with 0.9% saline epidural administration same to group Pro. In group Iso+Epi, isoflurane was provided combined with 0.5% ropivacaine epidural administration same to group Pro+Epi. Vasoactive drugs should be administrated when necessary to keep the fluctuation of blood pressure within 10 % of the baseline value.

Arterial blood and mixed venous blood were taken for blood gas analysisat following time points: before induction in supine position (T_1_), 30min after bilateral lung ventilation (T_2_), 15min after one lung ventilation (T_3_), 30min after one lung ventilation (T_4_), 60min after one lung ventilation (T_5_), 180min after one lung ventilation (T_6_), intrapulmonary shunt (Qs/Qt) was calculated according to the correlation formula. Qs/Qt=(Cc’O_2_-CaO_2_) / (Cc’O_2_-CvO_2_)*100%,CaO_2_=(Hb*1.31*SaO) + (PaO_2_*0.003), CvO_2_=(Hb*1.31*SvO_2_)+(PvO_2_*0.003), inhalation of 100% O2, Cc’O_2_=(Hb*1.31*SaO_2_) + (713-PaCO_2_/0.8)*0.003. (Cc’O_2:_ pulmonary capillary oxygen content, CaO_2_: arterialoxygen content, CvO_2_: mixed venous oxygen content, SvO_2_: mixed venous oxygen saturation, SaO_2_: mixed venous oxygen saturation, PvO_2_: mixed venous oxygen saturation. cardiac index (CI), mean arterial pressure (MAP), heart rate (HR) were recorded at the time points of T_1-6_.

### Statistical analysis

Data were analyzed using Statistical Package for the Social Sciences (SPSS) 20.0 (IBM). Chi-square test and two ways ANOVA were used to compare quantitative and categorical outcome. Independent sample t-test, Chi-square test were used to compare baseline subject characteristics. Unless otherwise noted, data are presented as the mean (SD) or frequency (%). Significance was defined as a two-sided *P*-value <0.05.

## RESULTS

There were no differences in demographic data, procedure length of the subjects (Table-I).

**Table-I:** Demographic data, procedure length of the subjects.

	Group Pro (n=20)	Group Pro+Epi (n=20)	Group Iso (n=20)	Group Iso+Epi (n=20)	
Gender (male/female)	12/18	15/15	14/16	17/13	17/13
Years	55±7	51±9	52±8	53±9	53±9
Weight (kg)	63±5	66±7	58±4	71±6	71±6
Length procedure (min)	220±30	265±28	238±26	315±35	315±35
ASA					
I	5	4	6	5	5
II	12	11	12	13	11
III	3	5	2	2	4

Qs/Qt values at T_2-6_ in four groups were significantly higher than that of T_1_, and Qs/Qt values at T_3-6_ was significantly higher than that of T_2_ (*P*< 0.05); PaO_2_ at T_2-6_ were significantly higher than that of T_1_, with PaO_2_ at T_3-6_ were significantly lower than T_2_ (*P*< 0.05). Between groups, Qs/Qt values in group Iso were significantly higher than that of group Pro, Pro+Epi and Iso+Epi at T3-5 (*P*< 0.05). There was no significant difference in PaO_2_ between groups (*P*> 0.05) (Table-II).

**Table-II:** Comparison of intrapulmonary shunt and oxygenation among the four groups (n=20).

Item	Group	T_1_	T_2_	T_3_	T_4_	T_5_	T_6_
Qs/Qt (%)	Pro	1.5±0.7	18.4±3.6^a^	29.2±3.3^ab^	34.1±4.8^ab^	29.2±3.8^ab^	29.3±24.4^ab^
	Pro+Epi	1.5±0.4	18.5±5.7^a^	27.1±3.5^ab^	27.4±7.2^ab^	29.3±4.7^ab^	26.5±1.7^ab^
	Iso	1.3±0.8	22.3±5.7^a^	45.8±13.7^abc^	41.0±14.8^abc^	42.5±11.5^abc^	34.5±4.8^abc^
	Iso+Epi	1.5±0.5	18.6±4.8^a^	34.2±3.6^ab^	32.8±7.1^ab^	32.5±7.4^ab^	29.1±8.9^ab^
PaO_2_ (mmHg)	Pro	83±12	390±64^a^	178±77^ab^	147±58^ab^	150±45^ab^	215±67^ab^
	Pro+Epi	85±10	425±54^a^	186±83^ab^	174±103^ab^	162±88^ab^	289±30^ab^
	Iso	85±11	423±58^a^	161±35^ab^	164±81^ab^	177±74^ab^	233±94^ab^
	Iso+Epi	82±10	419±84^a^	173±92^ab^	146±68^ab^	170±72^ab^	244±91^ab^

Compared with T_1_,

a P<0.05, Compared with T_2_,

b P<0.05, Compared with group Pro, Pro+Epi, Iso+Epi,

c P<0.05

CI at T_3-6_ in group Iso and Iso+Epi were significantly higher than that of T_1_ (*P*<0.05), and were significantly higher than that of propofol group (*P*<0.05). MAP at T_3-6_ in group Pro+Epi and Iso+Epi were significantly lower than that at T_1_(*P* <0.05). Heart rate at T_4-6_ in group Iso were significantly higher than T_1_, and higher than group Pro and group Iso+Epi (*P* <0.05) (Table-III).

**Table-III:** Hemodynamic changes of four groups at different time points.

	Group	T_1_	T_2_	T_3_	T_4_	T_5_	T_6_
CI (L/min/m^2^)	Pro	3.1±0.9	2.8±0.7	2.8±0.8	2.6±0.9	2.8±0.8	3.0±0.7
	Pro+Epi	3.2±0.3	3.3±0.4	3.1±0.5	3.0±0.6	2.5±0.8	3.0±0.5
	Iso	3.1±0.5	3.2±0.8	3.6±0.6^ab^	3.7±0.7^ab^	3.6±0.7^ab^	3.4±0.5^ab^
	Iso+Epi	3.0±0.5	2.8±0.6	3.4±0.5^ab^	3.3±0.8^ab^	3.5±0.6^ab^	3.5±0.5^ab^
MAP (mmHg)	Pro	96±8	90±10	92±11	89±13	97±14	93±11
	Pro+Epi	95±10	87±8	81±10^ac^	83±8^a^	82±8^ac^	85±10^ac^
	Iso	87±16	95±18	98±16	92±14	89±12	93±12
	Iso+Epi	92±8	79±12	72±10^ac^	70±9^ac^	68±12^ac^	74±14^ac^
HR (B/min)	Pro	76±15	71±16	72±14	76±16	72±14	75±16
	Pro+Epi	73±12	69±11	68±10	68±8	70±11	72±12
	Iso	82±15	78±16	85±16^bc^	90±18^abc^	89±17^abc^	88±16^abc^
	Iso+Epi	80±16	79±9^b^	72±12	78±11^b^	79±14	78±16

Compared with T_1_,

a P<0.05; group Iso vs. Pro, group Iso+EPi vs. Pro+Epi,

b P<0.05; groupIso vs. Iso+Epi, group Pro vs. Pro+Epi,

c P<0.05;

## DISCUSSION

We found that intrapulmonary shunt increased from initiation of one lung ventilation and reached a peak at 30min after initiation of one lung ventilation, then gradually decreased for the influence of HPV. There was no difference in intrapulmonary shunt until 180 minutes after one lung ventilation. Previous study also came to the same conclusion that influence of HPV improve the intrapulmonary shunt and oxygenation.^5^,^7^ Changes of PaO_2_ were contrary to the intrapulmonary shunt rate. When the shunt rate reached the highest, PaO_2_ decreased to the lowest, and then increased gradually. Previous studies have shown that PaO_2_ decreases to the lowest level at about 30min after one lung ventilation, after that oxygenation index (PaO_2_/FiO_2_) will increase gradually, and oxygenation will gradually be improved. However these changes are not associated with anesthesia methods.

We found the incidence of intrapulmonary shunt was higher in patients undergoing isoflurane based anesthesia than patients undergoing propofol based anesthesia, and the shunt rate in the isoflurane group was also significantly higher than that in the isoflurane combined epidural group. We may come to a point that compared with intravenous propofol based anesthesia, isoflurane inhalation based anesthesia has a greater impact on intrapulmonary shunt. Cho, Youn Joung, et al.^5^ demonstrated that vascular diameter in ventilated lung was significantly smaller under intravenous propofol anesthesia, erythrocyte velocity were significantly decreased; as contrast, pulmonary artery diameter only slightly decreased under isoflurane anesthesia. This phenomenon indicates that isoflurane may increase intrapulmonary shunt and reduce the systematic oxygenation.^8^,^9^

Reasons for increase of intrapulmonary shunt under isoflurane anesthesia mainly include the following:

1. Dilatation of small pulmonary arteries which inhibited the effect of HPV^10^
2. Increase of cardiac output and intrapulmonary shunt. Although isoflurane may increase intrapulmonary shunting, it does not significantly reduce PaO_2_;^9^,^11^,^12^

The reason might be that isoflurane increases cardiac output during one lung ventilation, thereby increases the blood flow at the ventilated side, improves the ratio of ventilation to blood flow, and improves the oxygenation.^13^,^14^

In our study, there was no significant difference in intrapulmonary shunt and PaO2 between patients with or without epidural block after 180 minutes one lung ventilation, which indicates that intrapulmonary shunt rate and oxygenation were not affected by epidural block. However, it should be noted that epidural block inhibited the sympathetic nerve, and further expanded the peripheral blood vessels, especially when combined with isoflurane inhalation, which might greatly enhance the effect of lowering blood pressure.^15^,^16^

### Limitations of the study

It was a relatively small trial and therefore easy to confounding from factors that we could not control. So these results need further validation. More work is required to define whether operations conducted laparoscopically or not will have an influence on the results.

*In* summary, intrapulmonary shunt, in patients undergoing one lung ventilation for 30 min, comes to a peak, with PaO_2_ to the lowest reading; then intrapulmonary shunt decreases and PaO_2_ increases gradually for the influence of HPV. Intravenous anesthesia with propofol takes on the least effect on the intrapulmonary shunt. Isoflurane inhalation can increase intrapulmonary shunt. Intravenous or inhaled anesthesia combined with epidural block does not affect intrapulmonary shunt and oxygenation, but attention should be paid to the influence of circulation.

### Authors’ Contributions

**MP:** Designed the study, managed the patients’ randomization preparation of the manuscript.

**ZL:** Helped to design the study, data analysis and writing the manuscript.

**XZ:** Responsible for collecting data and postoperative clinical assessment helped to write and revised the manuscript.

**KY:** Did anesthesia care, revised the manuscript.

All authors have approved the final version to be published.
